# Homophilic organization of egocentric communities in ICT services

**DOI:** 10.1371/journal.pone.0325187

**Published:** 2025-06-05

**Authors:** Chandreyee Roy, Hang-Hyun Jo, János Kertész, Kimmo Kaski, János Török

**Affiliations:** 1 Department of Computer Science, Aalto University School of Science, Espoo, Finland; 2 Department of Physics, The Catholic University of Korea, Bucheon, Republic of Korea; 3 Department of Network and Data Science, Central European University, Vienna, Austria; 4 Department of Theoretical Physics, Institute of Physics, Budapest University of Technology and Economics, Budapest, Hungary; 5 HUN-REN-BME Morphodynamics Research Group, Budapest University of Technology and Economics, Budapest, Hungary; University of Glasgow, UNITED KINGDOM OF GREAT BRITAIN AND NORTHERN IRELAND

## Abstract

Members of a society can be characterized by a large number of features, such as gender, age, ethnicity, religion, social status, and shared activities. One of the main tie-forming factors between individuals in human societies is homophily, the tendency of being attracted to similar others. Homophily has been studied mainly in the context of link formation and social dynamics. However, less is known about the role of the multidimensional homophily in forming egocentric communities on Information and Communications Technology (ICT) services. To close this gap, we analyze three ICT datasets, namely, two online social networks and one network deduced from mobile phone calls, in all of which data about individual features are available. We identify communities within egocentric networks and surprisingly find that the larger the community, the more overlap is found between features of its members and the ego. We interpret this finding in terms of the effort needed to manage the communities; the larger diversity requires more effort such that maintaining a large diverse group may exceed the capacity of the members. As the ego reaches out to their alters on an ICT service, we observe that the first alter in each community tends to have a higher feature overlap with the ego than the rest. Moreover, the feature overlap of the ego with all their alters displays a non-monotonic behavior as a function of the ego’s degree. We propose a simple mechanism of how people add links in their egocentric networks of alters that reproduces the empirical observations and shows the reason behind non-monotonic tendency of the egocentric feature overlap as a function of the ego’s degree.

## Introduction

One of the central questions in sociology is how social networks of individuals and linking ties between them are organized. These ties and their strengths are known to stem from the kinship [1], triadic closure [2, 3], and also to a large extent from homophily [4], that is, the tendency for similar individuals to become associated with each other. This association is based on demographic or sociological features of individuals such as race, gender, age, location, level of education, socioeconomic status, and hobbies. The homophilic association has been studied using a single feature, e.g., age [5], gender [6, 7], and race [8, 9], as well as using a combination of multiple features [10–16] called multidimensional homophily [14].

The multidimensionality of features can be translated into overlapping communities of social networks [17–21], implying that each individual may belong to multiple communities, each of which can be associated with one or more features. For example, a person is a member of a family, a company, and a hobby club at the same time. To investigate the interplay between the community structure and the multidimensional features, we focus here on egocentric networks [22, 23], consisting of an ego and alters, and linking ties between them. As for the structure of egocentric networks, and according to the Social Brain Hypothesis by Dunbar, humans or egos have on average around 150 active social contacts with others or alters [24–26]. Moreover, these 150 alters are not equally important or “emotionally close" to the ego. According to Dunbar [24], they are organized in hierarchical layers with sizes as ~5 closest ones, next ~15 close friends, followed by ~50 friends and social acquaintances.

Here we take an alternative approach for the egocentric networks by leveraging information of the features the alters are expected to share with an ego as a homophilic association. In addition, the alters may also form communities corresponding to different types of group to which the ego belongs. Such communities surrounding the ego will here be called egocentric communities. After identifying egocentric communities, one can investigate, for example, how many features are shared between an ego and alters in each community, in terms of the feature overlap. Such a feature overlap plays an essential role in many social models like the Axelrod model [27–29], social diffusion model [30], and homophily-based social network model [21, 31]. We find that the larger the community, the more overlap there is between features of its members and the ego, which is consistent with empirical results from voluntary organizations [32]. It also turns out that the feature overlap of the ego with all their alters displays a non-monotonic behavior as a function of the ego’s degree.

In this study, we analyze three datasets of Information and Communications Technology (ICT) services, namely, two online social network (OSN) sites and one network deduced from mobile phone calls, in all of which data about individual features are available. It should be noted that not all real social ties between users in ICT services appear in the corresponding datasets, implying that the egocentric networks of users are partial and sampled. Therefore, the observed network properties could be different from those of the real social network due to the sampling process, in other words, the channel selection mechanism [33, 34]. Despite the datasets being incomplete, we can still study the interesting question of the order of alters reached or connected by the ego in ICT services. This is possible since the snapshot of the social network site at a given time reveals in which orders the ego adds their friends. In particular, we find that as the ego reaches out to their alters on an ICT service, the first alter in each community tends to have a higher feature overlap with the ego than the rest.

This paper is organized as follows. We first discuss three ICT datasets to be analyzed and define measures quantifying the feature overlap between the ego and the alters. Next, we present our empirical findings for one of the three datasets, and devise a simple model to qualitatively reproduce the empirical findings. Finally, we conclude our work with discussions and present the results of two other datasets for comparison purposes.

## ICT data

The datasets we have access to and study are (i) iWiW, a Hungarian Online Social Network (OSN) [35], (ii) Pokec, a Slovakian OSN [36], and (iii) mobile phone data from a European country [37]. The first two datasets contain user-acknowledged friendship links, while the third one is created by aggregating Call Detail Records (CDRs). The iWiW was once the most popular OSN service for three years in Hungary; it had 4.6 million users, and an average degree of 208. The Pokec had similar success in Slovakia with 1.6 million users and an average degree of 27.3. The mobile phone data is from a provider in a European country with 5.7 million users. All three anonymized datasets are accompanied with information on individual features as summarized in Table 1.

**Table 1:** Feature statistics of the available datasets. Features are mostly self-declared and their availability is shown in percentage of all users in each dataset. The location refers to city for iWiW and one of the 188 Slovakian regions for Pokec. Features marked with ‘cat’ are categorical variables and those marked with ‘num’ are numeric variables.

Feature	iWiW		Pokec		CDR	
Age	61%	num	61%	num	86.5%	num
Gender	100%	cat	100%	cat	86.5%	cat
Location	89%	cat	100%	cat		
Education level	53%	cat				
Body Mass Index (BMI)			40%	num		
Alcohol consumption			48%	cat		
Sexual preference			31%	cat		
Smoking			54%	cat		

**Fig 1:**
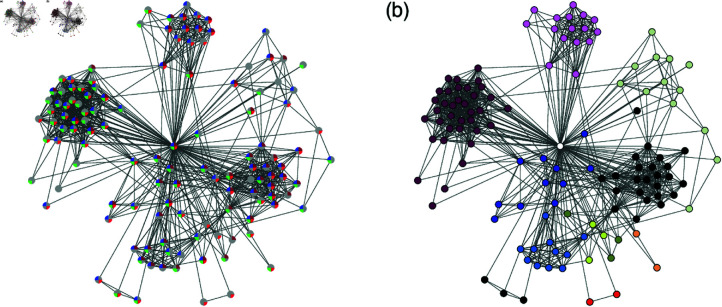
An exemplary egocentric network from the iWiW data. Links between the ego (central node) and their alters as well as between the alters are shown. (**a**) Small circles with four sectors represent the nodes and the four features available in the data. Whenever an alter has the same trait as the ego for a feature, that sector of the alter is colored as the ego (gender in red, city in green, education level in blue, and age in brown). Otherwise, if the alter’s trait does not match that of the ego, it is colored gray. (**b**) The same egocentric network in (a) is shown with communities detected by Infomap method [38] leaving out the ego node, where different communities are denoted by different node colors.

Each of this dataset contains several features of users, and each feature may have different values or traits. For example, there are two traits, “male” and “female,” allowed for the feature “gender". A feature is considered overlapping for a pair of connected users if those users’ traits for that feature match each other. For some features having continuous values of traits, we allow for value differences, e.g., we consider that two users are of the same age if the difference in their birth dates is less than two years. As an illustration of the role of the features we present a sample from the iWiW data in Fig 1. An egocentric network, i.e., the induced subgraph of an ego and their alters is shown, demonstrating the feature overlap. The traits of the four features of the ego are shown with different colored circle sectors. If a trait of an alter matches that of the ego, it is plotted with the same colored circle sector, otherwise if the alter’s trait does not match that of the ego (e.g., the alter lives in a different city), then that sector is colored gray. We observe that there are very few alters who do not have any matching feature with the ego and a likely explanation for this could be that those are relatives of the ego. It is worth noting that alters in different communities mainly have different sets of features in common with the ego.

Fig 1a was plotted using the Fruchterman-Reingold force-directed algorithm [39], which pulls the connected nodes together while keeping the nodes at distance. Thus, communities of strongly connected nodes become visible. We identify communities in the egocentric network using the two-level Infomap method [38] after removing the ego. We assume that communities found this way are also relevant in the ego’s real life [40, 41]. In Fig 1b it can be observed that the nodes in the largest community have a surprisingly large number of matching features.

The example in Fig 1 illustrates phenomena that have been observed by other researchers, namely that acquaintances of humans form communities [17] and that there is a strong homophily among them [5, 6, 10, 12]. The quantification of this would answer the question to what extent homophily is important in egocentric communities and what its consequences are. In order to quantify the similarity between users we introduce a similarity measure which is just the inverse of a Hamming distance [42]. The next questions we want to answer are what role the similarity plays in our choice of connecting to friends in ICT services and which factors drive our choices.

## Measures

In order to quantify the similarity of an ego to their alters in terms of the features, we first define a *feature overlap* function $\Delta (\sigma_{i}^{f},\sigma_{j}^{f})$ [21, 27]. This function has a value of 1 for matching traits of the *f*th feature between two connected users *i* and *j*, and a value of 0 for mismatching traits. If the feature is denoted by a categorical variable such as gender or city (marked with ‘cat’ in Table 1), the function Δ results in 1 only when traits of two users match exactly (it is then equivalent to the Kronecker delta). If the feature is a numeric variable of traits (marked with “num” in Table 1), then we allow the following difference in the matching feature: ±2 years difference for age and $\pm 1{kg/m}^{2}$ precision for BMI.

We define the *link feature overlap* between users *i* and *j* by the mean of the feature overlap between them only using the features that exist for both users:

$$o_{ij}\equiv \frac{1}{\left|F_{ij}\right|}\sum_{f\in F_{ij}}\Delta (\sigma_{i}^{f},\sigma_{j}^{f}),$$ (1)

where *F*_*ij*_ is the set of features available for both users *i* and *j*, and |*F*_*ij*_| is the cardinality of the set, in other words it is the number of features both *i* and *j* have. Only for the mobile phone data it may happen that *F*_*ij*_ is an empty set, in which case these users are removed from the analysis.

We characterize the similarity of an ego, say *i*, to a subset Λ of their alters in terms of the *subset feature overlap* which is an average of *o*_*ij*_ in Eq 1 for j∈Λ:

$$o_{i}(\Lambda )\equiv \frac{1}{\left|\Lambda \right|}\sum_{j\in \Lambda }o_{ij}.$$ (2)

Here we consider two cases for the subset Λ: (i) $\Lambda =N_{i}$, i.e., all alters of the ego, and (ii) $\Lambda =C_{i}^{r}$, i.e., alters in the *r*th egocentric community of the ego *i*. After determining the communities we focus on the average value of subset feature overlaps in Eq 2 over all communities of size *s* for all egos:

$$\langle o\rangle (s)\equiv \langle o_{i}(C_{i}^{r})\rangle_{\left\{i,r|s=|C_{i}^{r}|\right\}},$$ (3)

which we call a *community feature overlap*. Similarly, we define an *egocentric feature overlap*, by taking an average of subset feature overlaps in Eq 2 for all egos having the same degree *k* as follows:

$$\langle o\rangle (k)\equiv \langle o_{i}(N_{i})\rangle_{\left\{i|k=|N_{i}|\right\}}.$$ (4)

Throughout our analysis the degree of a node will be of crucial importance. It was already shown in Ref [33] that the degree of a node in an OSN is not determined by its number of friends in real life but rather, it might be the result of the inherent sampling of the real social network by the OSN. Moreover during the lifetime of the OSN the degree of a user tends to gradually increase. Therefore, we assume that the friends of a user in the datasets analyzed are a subset of its real life counterpart. But this subset is selected in a specific order characterized by the importance of the nodes to our social life.

Regarding the appearance order of alters in egocentric networks, we first chronologically sort out alters in each egocentric community for all egos; if an alter *j* in an egocentric community $C_{i}^{r}$ of size *s* appeared as the *n*th, the *j*’s appearance order is denoted by $a_{i}^{r}(j)=n$ for n=1,…,s. Then one can define an *appearance-order feature overlap* between egos and their *n*th appeared friends in all egocentric communities of size *s*:

$$\langle o\rangle_{s}(n)\equiv \langle o_{ij}\rangle_{\left\{i,j|s=|C_{i}^{r}|\text{and}a_{i}^{r}(j)=n\right\}}.$$ (5)

## Empirical results

In this Section we report empirical results only for the iWiW data, while the other two datasets are discussed later.

**Fig 2:**
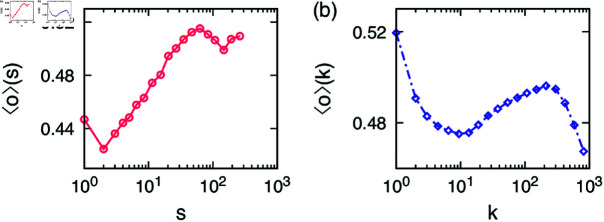
Empirical results of the iWiW data. (**a**) The community feature overlap as a function of community size ⟨o⟩(s) in Eq 3 and (**b**) the egocentric feature overlap as a function of the ego’s degree ⟨o⟩(k) in Eq 4. Standard errors are not shown as they are smaller than the symbol size.

One of the important features of egocentric networks is their community structure, as it forms the basis of our social life and represents various facets of our lives. In our sample egocentric network in Fig 1 we can see that some communities have more overlap with the ego than others and here we would like to quantify this observation. In the case of iWiW data, we could identify the users who had a reliable representation of their real social network [33]; we choose users with at least 7 years of activity or with the number of alters between 150 and 250 to perform the community detection using Infomap method [38]. Then we calculate the community feature overlap as a function of the community size *s*, i.e., ⟨o⟩(s) in Eq 3. In Fig 2a the result shows an increasing trend up to s≃50, after which the curve slightly decreases. Such an increasing tendency has been empirically found using the face-to-face contact data [32]. The results are robust with respect to the choice of users with any degree or any degree range. We remark that to test the robustness of our results, we have analyzed data using two other commonly-used community detection methods, i.e., the Louvain method [43] and the link-community detection method [18] (but only for around 1% of egos), leading to find the same tendency that the feature overlap increases with the community size.

In Fig 2b we show the egocentric feature overlap as a function of the ego’s degree *k*, i.e., ⟨o⟩(k) in Eq 4. We find a local minimum at k≃10 and a local maximum around at k≃200. The feature overlap around k≃200 has a value of ≃0.5 that is comparable to the average feature overlap found in some large egocentric communities.

We note that the features available in the data might be correlated with each other, biased at certain values (e.g., more users living in bigger cities) [44], and/or in different levels of missing data. Therefore, we scrutinize how robust our empirical findings are with respect to different settings. For this, we repeat the analysis using only one feature while ignoring other features so that the results may be free from possible correlations between features. We find that the results using only one feature show the qualitatively the same behaviors as those in Fig 2. In particular, ⟨o⟩(k) decreases to k≃10 then increases up to a maximum which is found between k≃80 for education level and k≃500 for cities. After that, the curves decrease for larger degrees.

In addition, we devise a randomized reference model [45] to find a baseline for the feature overlap. This is done by keeping the feature vectors, that is, $\left\{\sigma_{i}^{1},\ldots ,\sigma_{i}^{F}\right\}$ for each user *i*, but assigning them randomly to the users. This keeps the feature correlations intact, but destroys the social correlations. Firstly, the feature overlap averaged over all links in the randomized reference model is ⟨o⟩≈0.21. Considering that the overall range of the feature overlap in Fig 2 is 0.42–0.52, our results are significant, which can be related to the induced homophily proposed in Ref [32]. Secondly, from the randomized reference model we measure ⟨o⟩(s) and ⟨o⟩(k) only using one feature while ignoring others to find that all curves are more or less flat, which is indeed expected for the randomized reference model. All these results indicate that our empirical findings in Fig 2 are robust and significant.

Our findings appear counter-intuitive for both ⟨o⟩(s) and ⟨o⟩(k). One would expect smaller groups to be potentially more uniform than large ones just for statistical reasons, as it is easier to find a handful of friends with social features similar to the ego than a few dozen matching ones. Moreover, naive thinking suggests that on average feature similarity correlates with emotional closeness between the pairs keeping in mind Dunbar’s layers of friendships [46] which states that relationships are highly structured and friendships are formed in layers with closest friends being in the innermost circle of an ego and least closest friends being in the outermost circle. Thus, if we reach our friends in the OSN in the order of emotional closeness, then no peak other than the one in *k* = 1 should be visible in the curves of ⟨o⟩(k).

**Fig 3:**
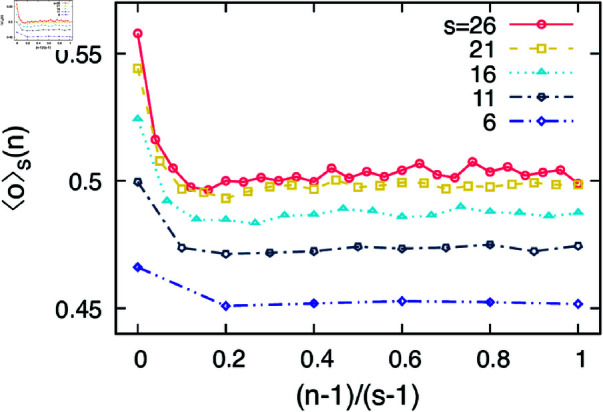
Empirical results of the iWiW data. We show the appearance-order feature overlap $\langle o\rangle_{s}(n)$ in Eq 5 for several values of *s*. Here the order *n* is scaled with the community size *s* for clearer visualization.

To study the above issue regarding the non-monotonic curves of ⟨o⟩(k) in more detail, we look inside the communities and check how the overlap is distributed for alters that appeared at different times. For this, we observe the evolution of egocentric networks in terms of the number of alters added; more precisely, we measure the appearance-order feature overlap $\langle o\rangle_{s}(n)$ in Eq 5. In Fig 3 we depict the feature overlaps of alters in the communities in the order of their appearance that are averaged over 25–160 thousand communities. The appearance order has been scaled by the size of the community for a clearer visualization. The curves for the larger communities are above the smaller ones as expected from Fig 2a. However, there is an interesting observation in the curves, namely that the first few alters have significantly higher overlap than the rest, having quite similar overlap values. It seems that in the egocentric communities only a few alters are really important for the ego reflecting the strong inhomogeneity of emotional closeness [46]. They are the ones with whom we connect to if we want to establish a community. Once the core of the community is created, the rest of alters are connected in a seemingly arbitrary order, i.e., irrespective of their feature overlaps with the ego.

Let us reiterate two important findings: (i) the feature overlap of alters in egocentric communities increases with the community size and (ii) the feature overlap of alters appeared in the community is unevenly distributed such that on average only the first one or two members are really close to us while the rest have less but almost uniform importance.

## Model

Let us devise a simple model that relates empirical observations on egocentric communities, i.e., ⟨o⟩(s) and $\langle o\rangle_{s}(n)$, to the non-monotonic behaviors of the egocentric networks, i.e., ⟨o⟩(k). Our model is neither for generating the entire social network nor for studying the role of structural mechanisms in egocentric networks. We rely on two observations: The first one is the observed ⟨o⟩(s) in Fig 2a which increases with increasing community size up to s≃50, after which it slowly decreases again. The second one is the observed $\langle o\rangle_{s}(n)$ in Fig 3. That is, each user adds friends one by one to its egocentric network by first choosing a community randomly, then from each community the first person has a higher feature overlap with the ego than the rest. The question we ask here is whether these ingredients are enough to recover the non-monotonic behavior of the egocentric feature overlap as a function of the ego’s degree, i.e., ⟨o⟩(k) in Fig 2b.

We first assume that the distribution of community sizes follows a power law with exponent –1.5. This exponent value is based on the analysis of iWiW data. More precisely, we assume that

$$P(s)=As^{-1.5}\text{for}2\leq s\leq 100,$$ (6)

with a normalization constant $A=1/(\sum_{s=2}^{100}s^{-1.5})$. Let us consider an ego whose real-world degree is $k_{\text{real}}$. Then we draw a number of community sizes from *P*(*s*) in Eq 6 such that the sum of those sizes equals $k_{\text{real}}$. Let the ego join an ICT service and start to make connections to alters from those communities one by one until the ego’s degree *k* in the service reaches $k_{\text{real}}$. Then, users of the service whose degree is *k* can be seen as egos who have sampled *k* alters from the real-world alters. In our model, we consider the cases with $k_{\text{real}}=150$ and 300.

**Fig 4:**
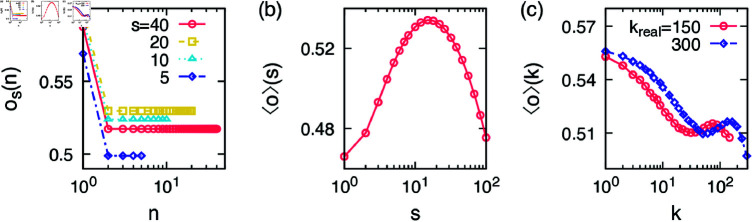
Model assumption and results: (**a**) Appearance-order feature overlap curves assumed for the model in Eq 7 for several community sizes *s*. (**b**) The resultant curve of ⟨o⟩(s) given by Eq 8. (**c**) Simulation results of the egocentric feature overlap as a function of the ego’s degree for the cases with $k_{\text{real}}=150,300$.

Next, we model the appearance-order feature overlap for alters in communities of the ego; for a community of size *s*, the ego’s *n*th alter in the community is assumed to have the feature overlap with the ego as follows:

$$o_{s}(n)=\left(1-\frac{1}{s+2}\right)\left(\frac{60}{s^{0.7}+100}\right)+0.07\delta_{n,1}\text{for}n=1,\ldots ,s,$$ (7)

where δ is a Kronecker delta. The first term on the right hand side of Eq 7 is an increasing and then decreasing function of community size *s* for the same *n*. It reflects the observation in Fig 2a. The second term on the right hand side of Eq 7 is to demonstrate the observation that the first appeared alter in each community tends to have the larger feature overlap with the ego than the rest, see Fig 3. Note that the functional form and the values chosen here are not fitted from the data but for demonstration purposes, since we only seek qualitative match with ⟨o⟩(k). Some typical functions are plotted in Fig 4a.

Using the appearance-order feature overlap of the model in Eq 7, we calculate the community feature overlap as

$$\langle o\rangle (s)=\frac{1}{s}\sum_{n=1}^{s}o_{s}(n).$$ (8)

The numerical result of Eq 8 is shown in Fig 4b, and it is similar to the empirical results in Fig 2a such that it increases for community sizes up to 10–20 and decreases afterwards.

Now let us introduce a model for the growing egocentric networks in an ICT service. We assume that people joining the service have a well established social network in their real life, where the friends can be sorted into disjoint communities. Since we focus only on the feature overlap between the ego and their alters not between those alters, what matters is the distribution of the feature overlap within communities not the internal structure of the communities. The ego joins the service as an isolated user.

1. A community, say *r*, is randomly chosen from the set of communities of the user.
2. Among remaining individuals in the community *r*, we take an individual having the largest feature overlap with the ego, which becomes the ego’s alter in the service.
3. That alter is removed from the list of available individuals in the community *r*. If all individuals of the community *r* are already connected to the ego, it is removed from the set of considered communities.
4. The above procedure (1–3) is repeated until the ego’s degree reaches $k_{\text{real}}$.

Using this procedure we generate 5,000 egocentric networks to take the average of the feature overlaps of the *k*th alters of all egos, i.e., ⟨o⟩(k), which is shown in Fig 4c. The curve ⟨o⟩(k) starts with a large value; the first alters have higher feature overlaps with the ego because each of them is the first appeared alter in each community. Then the curve decreases as the rest of alters in communities who have lower values of the feature overlap start to be added to the egocentric network. Once alters in smaller communities with smaller feature overlap are all added, then only alters in larger communities are left. Thus for larger *k* we find the increasing tendency of the ⟨o⟩(k) curve. Finally, when the nonsocial alters in very large communities are added to the egocentric network, ⟨o⟩(k) decreases again. This is why we have both local minimum and local maximum in the ⟨o⟩(k) curve.

The model introduced here reproduces qualitatively the empirical findings, but the assumption that the communities are chosen uniformly at random needs to be further tested. Consider an ego whose egocentric network has *c* communities. We can track the chronological order of alters appeared in the egocentric network and which community each alter belongs to. That is, we know the chronological order *m* of the alter who appeared in the egocentric network for the first time among members of each community, leading to exactly *c* values of *m* for each ego. By collecting such *m*s for all egos whose egocentric networks have *c* communities, we get the probability distribution function *P*_*c*_(*m*). If our assumption that communities are randomly chosen is correct, we expect to have

$$P_{c}(m)={\left(1-\frac{1}{c}\right)}^{m-1}\frac{1}{c}\approx \frac{1}{c}e^{-m/c},$$ (9)

where the approximation has been made by assuming that m,c≫1.

**Fig 5:**
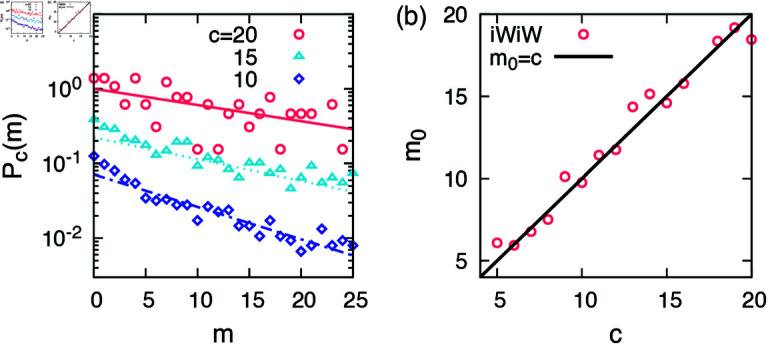
Empirical results of the iWiW data. (**a**) We show probability distribution functions *P*_*c*_(*m*) of the chronological order *m* of the alter who appeared in the egocentric network for the first time among members of each community for egos having *c* communities. Theoretical expectations of $P_{c}(m)\propto e^{-m/c}$ are shown in lines. Empirical results and expected curves for *c* = 15 and 20 are shifted for clearer presentation. (**b**) Estimated values of the scale parameter *m*_0_ in Eq 10 as a function of *c*. For comparison, the expectation *m*_0_ = *c* by Eq 9 is plotted in black line.

The distribution *P*_*c*_(*m*) can be directly measured from the iWiW data as the chronological orders of alters of given egos and communities alters belong to are available. As shown in Fig 5a the distribution *P*_*c*_(*m*) turns out to be exponential for the range of m≤25 for several values of *c*, implying that our assumption is reasonable. By assuming that

$$P_{c}(m)\propto e^{-m/m_{0}}$$ (10)

with a scale parameter *m*_0_, we estimate the value of *m*_0_ for each value of *c*. It is expected that *m*_0_ = *c* by Eq 9. The results are depicted in Fig 5b, showing that our expectation *m*_0_ = *c* is indeed the case. Note that communities have finite number of members and once all members in a community are added to the egocentric network, then that community cannot be selected any more. Thus *P*_*c*_(*m*) becomes no more exponential. This is why we fit the empirical distribution for the range of m≤25 to obtain convincing exponential functions.

## Discussion

By analyzing empirical data from the iWiW, we have shown that on average, the ego has larger feature overlap with alters in the larger egocentric communities. At first, this result might be surprising since it is always more difficult to find more people who are similar to us. Thus, populating a large community with very similar friends would seem more difficult than a smaller one. However, we present some arguments for these observations. Social links fade over time, and they need regular interactions to remain active [47]. Thus, in order to maintain many links within a large community more activity is needed, which in turn requires more social similarity [21, 27].

We have also shown that as egos populate their egocentric networks with the alters, they choose the communities apparently in random order. Then among members of each community the ego tends to first connect to a few members who are most similar to the ego, and other members who are connected later to the ego show homogeneous but smaller similarity to the ego. This finding, together with an increasing feature overlap with larger egocentric communities, is found to be consistent with the egocentric feature overlap that has a local minimum at around *k* = 12–15, which is very close to the size of the Dunbar’s layer for close friends. It strongly implies that egos make connections to their close friends not from a single community but from multiple communities with diverse backgrounds, hence showing a local minimum of the egocentric feature overlap. Thus, connections to those close friends might represent the backbone of the egocentric networks. For large degrees, the egocentric feature overlap shows a decreasing trend after ~200 due to the increasing fraction of nonsocial contacts. The number 200 is close to the Dunbar’s number. Our data indicates that indeed after the aforementioned limit the nature of the ties changes.

Finally, we discuss a future work based on the possibility that egocentric communities are overlapping, implying that some alters of the ego may belong to the same communities simultaneously. Since the Infomap community detection method [38] leads to only non-overlapping communities, it should be worth analyzing the data in more detail based on overlapping community detection methods [18].

## Other datasets

### Pokec data

Pokec is an existing Slovakian social network site. The dataset [36] contains profile information for some 1.6 million users for a country with 5.5 million inhabitants. It also includes connection data, which are not time-stamped. The profile contains many information fields. We used the ones for which at least 30% of the users gave meaningful answer. Some fields such as alcohol, sex, smoking related habits were free strings. There were 3–5 answers with identical strings which were enumerated in our work. For examples, we considered ‘abstinent’ as it is, “pijem prilezitostne” as “I drink occasionally,” ‘pijem pravidelne’ as “I drink regularly,” “pijem” as “I drink.” The BMI was calculated from the given height and mass values and only reasonable values between 10 and 50 were kept. The location was given by counties. The two major cities, Bratislava and Košice, are divided into sub regions, implying that these were not aggregated into one city.

**Fig 6:**
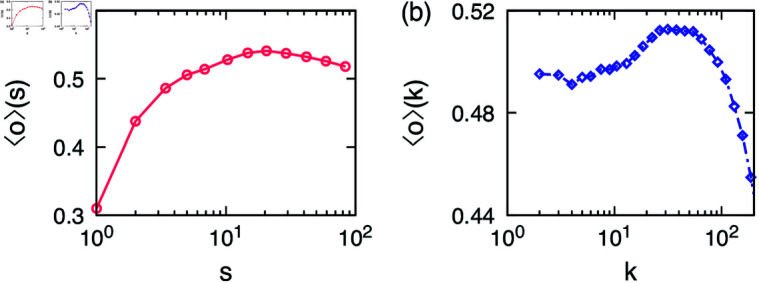
Empirical results of the Pokec data: (**a**) the community feature overlap as a function of community size ⟨o⟩(s) in Eq 3 and (**b**) the egocentric feature overlap as a function of the ego’s degree ⟨o⟩(k) in Eq 4.

The community feature overlap is shown in Fig 6, showing that the curve increases up to s≃20, which is comparable to the behavior of the iWiW data which has three times more users and average degree than Pokec. The egocentric feature overlap in Fig 6b shows a maximum at around k≃30–50. The initial peak is only marginal.

### Call detail records

In addition to the OSN datasets, we analyze the mobile phone dataset that has 5,716,084 subscribers from a European country in the year 2007 from January to July whose demographic information is displayed in Table 1. The users were anonymized by the service provider with unique identifiers such that the privacy of the individuals are protected and cannot be traced back to them. The call detail records (CDRs) include all the incoming and outgoing calls made by an individual during the study period. It also includes the age and gender of the caller and the callee which have been considered as the main features for feature overlap calculation in the present paper. We studied approximately 600,000 egocentric networks sampled randomly from the data and filtered out possible call centers or business centers by making sure that the total number of alters did not exceed 1000 for each ego. We considered only those pairs that have mutually reciprocated at least one call or SMS in the study period in the egocentric network for calculation of the feature overlap between the pairs. We were left with 123,533 egocentric networks comprising 482,309 users (222,312 females and 259,997 males) with the egos and their alters having at least one known feature. The age of the egos has a right-skewed distribution for both genders with the mean, median and mode being 42.5, 40 and 32, respectively.

**Fig 7:**
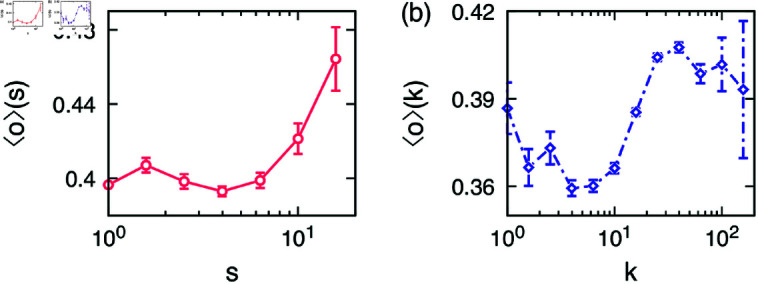
Empirical results of the CDRs: (**a**) the community feature overlap as a function of community size ⟨o⟩(s) in Eq 3 and (**b**) the egocentric feature overlap as a function of the ego’s degree ⟨o⟩(k) in Eq 4. The error bars represent standard errors.

In Fig 7a we show the community feature overlap as a function of community size. As shown in the figure, the range of community size is limited due to the nature of CDRs analyzed here. In our work, the link between two users is considered meaningful only when they have called each other at least once during the period of study. Thus the average degree itself is quite small, leading to relatively small egocentric community sizes, in contrast to iWiW and Pokec datasets. We also note that since it requires an extra effort to make calls and talk using mobile phones we believe that the network from CDRs consists of more meaningful relationships, for example, important friends and family members, who are called frequently and on a regular basis. Moreover, the study period was considered for the year 2007 when mobile phones were still being used as a major form of communication channel, and therefore this network represents fewer but closer relationships. However, we find a similar tendency of the community feature overlap to the OSN datasets, namely, the community feature overlap increases from a community size 4 onward. Fig 7b shows the egocentric feature overlap. Here, we considered all those neighbors whose at least one feature is known. We find the same patterns as the iWiW and Pokec datasets. These patterns disappear when we shuffle the traits or features of the alters. Additionally, if we consider only those pairs in the networks that have high call/SMS activity, then the values of overlap become greater because the pairs that are more talkative indicate an even closer relationship among them. However, the patterns of overlap are still found to be the same.

## Summary

In summary we have shown by analyzing three different empirical data sources that the larger the egocentric community the more similar its members are to the ego. As a user adds its friends to its egocentric network on an OSN the communities appear essentially in a random manner. We have also shown that in all egocentric communities we first connect to one or two really close friends while the rest on average has comparable emotional closeness. Finally we tested our empirical findings by devising a simple model that can relate all the mentioned empirical findings in a consistent way.
